# A Case Report: Anti-Psychotic Agents Related Ocular Toxicity

**DOI:** 10.1097/MD.0000000000003360

**Published:** 2016-04-18

**Authors:** Bonnie Nga Kwan Choy, Alex Lap Ki Ng, Jennifer Wei Huen Shum, Michelle Ching Yim Fan, Jimmy Shiu Ming Lai

**Affiliations:** From the Department of Ophthalmology (BNKC, ALKN, JWHS, JSML), LKS Faculty of Medicine, The University of Hong Kong; and Department of Ophthalmology (MCYF), Queen Mary Hospital, Hong Kong SAR, People's Republic of China.

## Abstract

Chlorpromazine is known to cause ocular pigmentary deposits. However, delayed presentation after cessation of chlorpromazine has not been reported. There are also no reports on whether newer generation of anti-psychotic agents contribute to ocular toxicity. We describe a case of ocular toxicity related to anti-psychotic agents. To the best of our knowledge, this is the first reported case of anterior segment pigmentary deposits associated with olanzapine use, 2 years after the cessation of chlorpromazine.

We report a case of ocular toxicity in a patient with history of chlorpromazine usage of 100 mg per day for 13 years and subsequently switched to olanzapine 5 mg for 2 years. There were no signs of ocular toxicity while the patient was on chlorpromazine. However, when the patient switched to olanzapine, she developed the ocular side effect as described for chlorpromazine-induced ocular toxicity, with pigmentary depositions on both corneas and the anterior lens surface and decrease in vision.

Olanzapine, a newer anti-psychotic agent, may play a role in the ocular pigmentary deposition, either directly causing pigmentary deposition itself or accentuating the effect of chlorpromazine as the 2 drugs act on the same receptors, although further studies are required to support this hypothesis. As patients with psychiatric conditions may not voluntarily complain of visual symptoms, ocular screening could be considered in these patients receiving chronic anti-psychotic treatment, so that any ocular toxicity could be diagnosed in a timely manner.

## INTRODUCTION

Anti-psychotic agents especially chlorpromazine are known to cause ocular pigmentary deposits. The effect is dose-dependent and it is not uncommon to identify anterior segment pigmentary deposits in the patients on chronic chlorpromazine treatment. However, delayed presentation after cessation of chlorpromazine has not been reported. Furthermore, there are emerging newer generations of anti-psychotic agents with increasing popularity due to their better safety profile, one of which being olanzapine. There are no reports on whether these newer generations of anti-psychotic agents contribute to ocular toxicity. In this case report, we described a schizophrenic patient who had been on chronic chlorpromazine without any noticeable ocular pigmentary deposits. She was noted to have anterior segment pigmentary deposits after chlorpromazine had been stopped for 2 years, while she is on olanzapine.

## CONSENT

This study adhered to the tenets of the Declaration of Helsinki. Informed consent was obtained from the patient for publication of this report and its related images.

## CASE PRESENTATION

**FIGURE 1:**
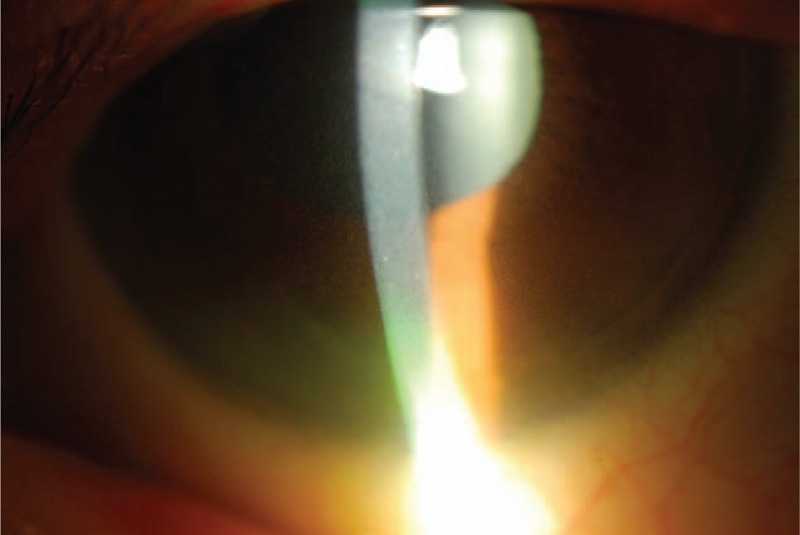
Diffuse corneal pigmentary deposits.

**FIGURE 2:**
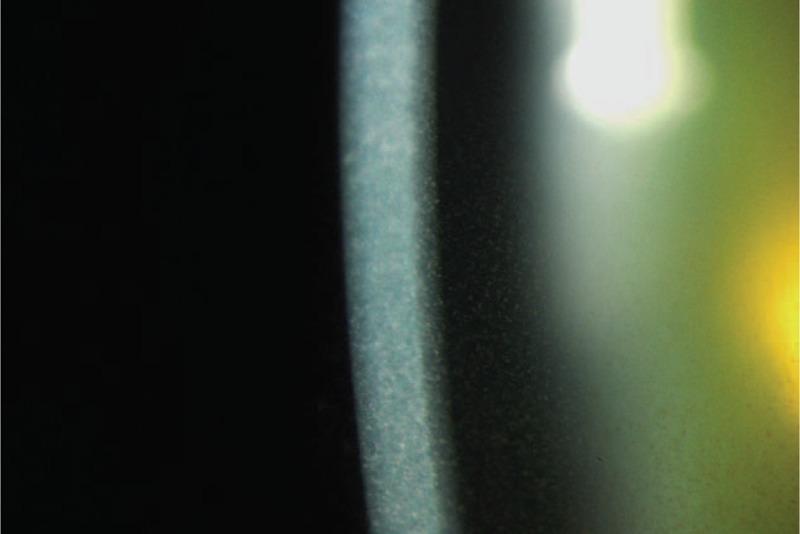
Magnified view showing granular pigmentary depositions from the anterior stroma to the endothelium of cornea.

**FIGURE 3:**
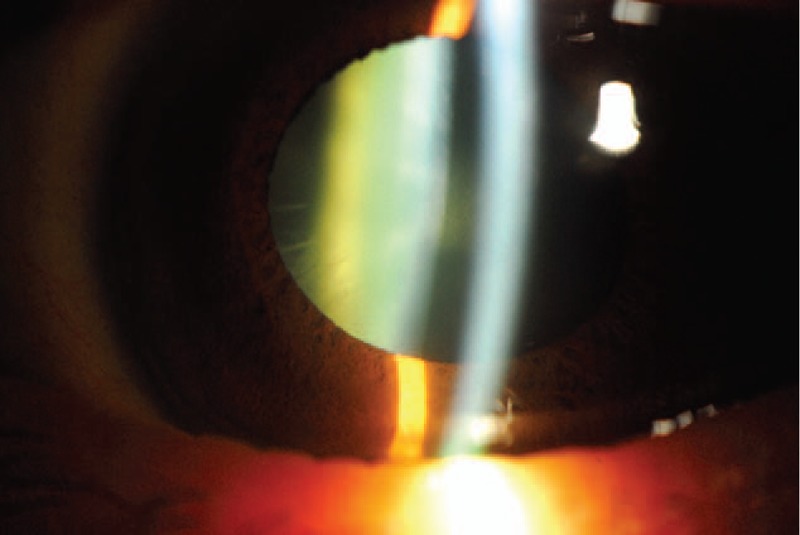
Pigmentary depositions over the anterior lens capsule.

**FIGURE 4:**
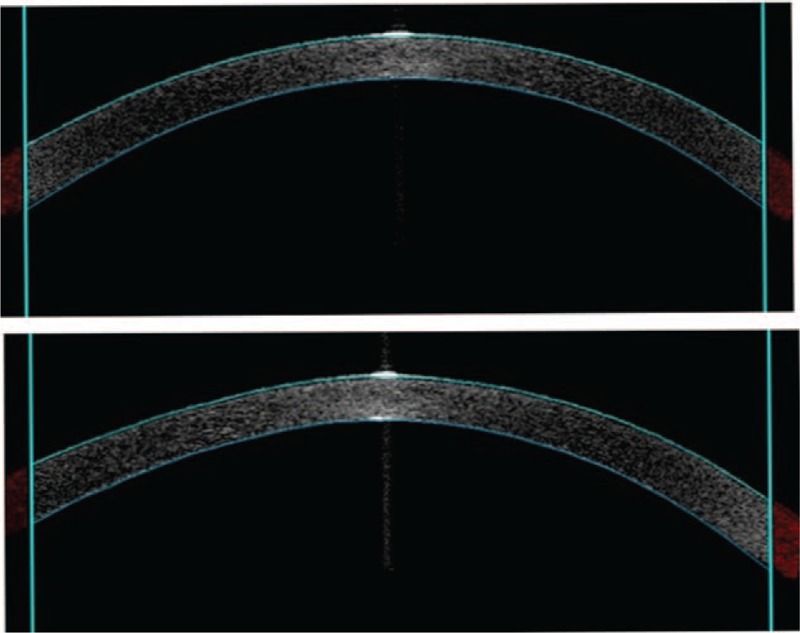
Anterior segment optical coherence tomography showing the presence of deposition involving the full thickness of cornea (above: right eye, below: left eye).

A 66-year-old woman with catatonic schizophrenia since 1972 was initially referred for blurred vision in 2012. She had been on chlorpromazine 100 mg daily for 13 years since 2000. On presentation, slit lamp and dilated fundal examination was unremarkable other than mild nuclear sclerosis cataract and the visual acuity was 0.3 (Snellen decimal) over both eyes. There were no abnormal corneal or lens capsular depositions and the cornea were clear bilaterally. The visual acuity remained the same for the following 2 years. In May 2013, chlorpromazine was switched to 5 mg daily olanzapine by her psychiatrist. She was not on any other systemic medications otherwise. In the latest follow-up in October 2015, there was a decrease in her visual acuity to 0.2 and 0.1 over her right and left eyes respectively. Slit lamp examination revealed bilateral diffuse pigmentary deposits over the full thickness of cornea (Figures 1 and 2), more significant at the interpalpebral region, as well as the anterior lens capsule (Figure 3), which was not present in the previous follow-up 6 months ago. There was no significant progression of her cataract. The pupils could not be fully dilated. Gonioscopy was attempted to assess for presence of deposits at the angle but the patient could not cooperate. Fundal examination was normal and no pigmentary retinal deposits were present. General inspection showed no cutaneous pigmentation. Anterior segment optical coherence tomography (Visante, Carl Zeiss Meditec, Dublin, CA) showed the presence of depositions involving the full thickness of the cornea (Figure 4). Specular microscopy revealed normal endothelial cell morphology, preserved hexagonal appearance, and the cell density was 3571 and 2958 over right and left eyes respectively. The clinical appearance of the corneal and lens deposition was classical of chlorpromazine-induced ocular toxicity, although the medication had been stopped for more than 2 years already when the ocular signs appeared. In view of the likely anti-psychotic agent related ocular toxicity, a letter explaining the ocular clinical findings was issued to her psychiatrist to reassess the need to continue the anti-psychotic agent. Olanzapine was subsequently stopped by her psychiatrist.

## DISCUSSION

Chlorpromazine has been known to cause ocular pigmentary depositions. This effect was first reported in 1964, where 70 patients on chronic chlorpromazine therapy for at least 3 to 5 years were found to have pigment deposition in skin, cornea, and lens capsule.^1^ Subsequently, there were a number of reports of chlorpromazine-related ocular pigmentary depositions, especially over the cornea and lens capsule.^2–4^ Chlorpromazine was also shown to be cytotoxic to corneal endothelium.^5^ Apart from chlorpromazine, other anti-psychotics that were attributed to causing ocular pigmentary deposits include levomepromazine,^6^ fluphenazine, trifluoperazine,^7^ and clozapine.^8,9^ There are suggestions that chlorpromazine binds to the serotonin and dopamine receptors in the cornea, resulting in corneal deposition.^10^ As olanzapine also has affinity to the serotonin and dopamine receptors, it may potentially exacerbate or even cause ocular depositions, which may explain the delayed presentation after cessation of chlorpromazine for 2 years.

In previous reports, subjects were found to have ocular pigmentary deposits during the course of chlorpromazine treatment. After cessation of chlorpromazine, there was report of irreversibility of the ocular pigmentary deposits,^11^ while other reported resolution of corneal deposition with time but not the lenticular deposition.^12^ In our patient, there was absence of anterior segment depositions despite chronic chlorpromazine therapy for 13 years. This suggests that the ocular pigmentary deposits in our patient may be related to olanzapine. As both chlorpromazine and olanzapine demonstrate affinity to serotonin and dopamine receptors, olanzapine can either lead to ocular toxicity by itself, or exacerbate the previous accumulative effects of chlorpromazine. To the best of our knowledge, both of these effects have not been reported in the literature. However, as our patient had been on chlorpromazine prior to the use of olanzapine, we would need further evidence from the patients on olanzapine alone to establish the causal relationship between olanzapine and its ocular toxic effects.

The exact mechanism of chlorpromazine-related ocular pigmentary deposition is unknown, but it is believed to be related to ultraviolet light-induced drug decomposition,^5^ as evident by the more common occurrence in the interpalpebral region. There was 1 case report in which a patient with unilateral ptosis demonstrated asymmetrical anterior segment pigmentation.^13^ As our patient lives in a hostel with little outdoor activities, it could explain the delayed presentation of ocular toxicity despite a chronic use of chlorpromazine for 13 years. Apart from the deposition of pigments, the inability for the pupils to be fully dilates suggested possible toxicity to the ciliary muscle.

To conclude, we reported a case with anterior segment pigmentary deposition after cessation of long-term chlorpromazine therapy and switching to olanzapine. Olanzapine, a newer anti-psychotic agent with increasing popularity, might exacerbate or even directly contribute to the pigmentary deposits by acting on the same receptors in the anterior segment as chlorpromazine, although the hypothesis requires further studies to support. As patients with psychiatric conditions may not voluntarily complain of visual symptoms, ocular screening could be considered in these patients receiving chronic anti-psychotic treatment, so that any ocular toxicity could be diagnosed in a timely manner. In case of established ocular toxicity, the responsible psychiatrists should be informed to evaluate if a change or even suspension of anti-psychotic agents is warranted to reduce further ocular toxic effects.
